# GPS-Based Network Synchronization of Wireless Sensors for Extracting Propagation of Disturbance on Structural Systems

**DOI:** 10.3390/s24010199

**Published:** 2023-12-29

**Authors:** Jesus Ricardo Salazar-Lopez, Jesus Roberto Millan-Almaraz, Jose Ramon Gaxiola-Camacho, Guadalupe Esteban Vazquez-Becerra, Jesus Martin Leal-Graciano

**Affiliations:** 1 Department of Computer Science, Autonomous University of Sinaloa, Culiacan 80013, Mexico; jesusricardo@uas.edu.mx; 2 Department of Physics and Mathematics, Autonomous University of Sinaloa, Culiacan 80040, Mexico; 3 Department of Civil Engineering, Autonomous University of Sinaloa, Culiacan 80040, Mexico; jesusleal@uas.edu.mx; 4 Department of Earth and Space Sciences, Autonomous University of Sinaloa, Culiacan 80040, Mexico; gvazquez@uas.edu.mx

**Keywords:** wireless sensor networks, pulse per second, synchronization, GPS, real-time clock, structural health monitoring, acceleration, disturbance propagation

## Abstract

Wireless sensor networks (WSNs) have gained a positive popularity for structural health monitoring (SHM) applications. The underlying reason for using WSNs is the vast number of devices supporting wireless networks available these days. However, some of these devices are expensive. The main objective of this paper is to develop a cost-effective WSN based on low power consumption and long-range radios, which can perform real-time, real-scale acceleration data analyses. Since a detection system for vibration propagation is proposed in this paper, the synchronized monitoring of acceleration data is necessary. To meet this need, a Pulse Per Second (PPS) synchronization method is proposed with the help of GPS (Global Positioning System) receivers, representing an addition to the synchronization method based on real-time clock (RTC). As a result, RTC+PPS is the term used when referring to this method in this paper. In summary, the experiments presented in this research consist in performing specific and synchronized measurements on a full-scale steel I-beam. Finally, it is possible to perform measurements with a synchronization success of 100% in a total of 30 samples, thereby obtaining the propagation of vibrations in the structure under consideration by implementing the RTS+PPS method.

## 1. Introduction

Nowadays, one of the most important philosophies used to assess the integrity of infrastructure is the well-known Structural Health Monitoring (SHM) approach [1]. Such a technique can be used to detect invisible or hidden damage that may be present in structures. Within this context, one of the main goals of the SHM philosophy is to detect unexpected changes in the main characteristics of structures, considering, in most of the cases, differences among the signals obtained by different sensors. As a result, the severity of the damage can be detected even in complex structural configurations [1]. In recent years, the SHM philosophy has been implemented in several structures around the world to prevent possible threats due to the inadequate structural condition that some structures, particularly the old ones, may be presenting. In general, the SHM of infrastructure can be implemented in different ways, and it can be defined as the detection and evaluation of damage in structures. Thus, the main objective of SHM can be established to be the estimation of structural damage to prevent a possible failure of structures, which may result in human and economic losses. Damage prevention by implementing SHM is essential since it provides safety in both construction and maintenance of structures. In this frame of reference, in the study published in [2], a comprehensive review of the SHM philosophy is carried out. In general terms, the current methods for structural monitoring and their advantages and disadvantages are reported. In addition, a particular emphasis is given to this aspect by considering the Internet of Things (IoT) approach. Finally, in this paper, the IoT technology is described as an option to establish communication between all of them, providing easy access to information of vital importance for SHM. Based on this discussion, it can be reported that SHM is utilized in several engineering disciplines such as civil, maritime, mechanical, military, etc. [3]. In the following paragraphs of this introductory section, a particular technical review on the SHM philosophy as well as its main implementations on the monitoring of several infrastructure systems are presented.

The dynamic response or vibration of structures generally propagates in the form of elastic waves, which implies that the study of wave propagation in structures can reveal the structural dynamic characteristics of the response and may provide the required information for proper structural design and control [4]. Based on what is reported in [5], in the SHM philosophy, there exist different techniques that can be used to detect damage in structures. In general, two procedures can be implemented for damage detection. These procedures are destructive and non-destructive tests, respectively. In [5], the authors highlighted the importance of tests that do not damage the structures and categorize the different techniques that exist to perform them. Some of the most popular sensors to register the dynamic response of bridges are GPS (Global Positioning System) devices and accelerometers. Both are widely used in the literature to compute the dynamic characteristic of structures and to predict possible damages on them. Thus, the integration of them in the form of a wireless node may represent an improvement to the SHM philosophy or an alternative to the usual instrumentation implemented in this philosophy. A significant benefit of implementing wireless nodes is their easy installation on structures whose configuration makes cable handling a real challenge.

The calculation of the dynamic response of structures using the GPS technology is still under development. Although several investigations on this topic exist [6], there are some drawbacks and problems such as multipath errors due to the presence of atmospheric effects. In addition, in most of the cases, it is necessary to use more than one GPS device to determine the position of a point if a millimeter precision is required. This is generally performed implementing the Real-Time Kinematic (RTK) technique. The implementation of this approach, alongside the use of GPS technology in SHM philosophy, has been widely reported in the literature [6,7,8,9,10,11].

The structural monitoring process of a bridge structure can be a complex task. It is mainly difficult because of the structural characteristics that they may present. For instance, some bridges present very long spans, which represents a real challenge to the installation of sensors and devices. In the case of cable-stayed bridges, acceleration sensors and the well-known strain gauges are generally installed on critical points of structures. Thus, installing wired sensors becomes a very difficult process. Given these scenarios, IoT wireless communication technologies have been developed. For example, in [12], the advantages of using wireless sensors on a railway bridge are discussed. The study presented in [13] describes the implementation of a complete IoT platform for a SHM procedure. The platform uses a combination of hardware and software to remotely collect, process, and transmit monitoring data from structures. This represents an opportunity for implementing alternative sensors to extract the structural response of bridges. As an option, the development of cost-effective wireless sensors, in the form of nodes using ZigBee radios with low power consuming piezoelectric sensors [14], represents a step in the right direction in SHM philosophy.

Recent wireless technologies such as LoRa and LoRaWAN have been developed for low power consumption and long-range communication. Furthermore, the LoRa protocol was utilized in [15], where a wireless monitoring system based on LoRa was developed, resulting in a cost-effective implementation. As an alternative to the above-mentioned studies, in this paper, different Wireless Sensor Networks (WSN) are implemented, mainly based on 915 MHz radio. These radios present a long range and minimize energy consumption simultaneously [16]. Thus, a unique energy autonomy can be achieved. Additionally, Wi-Fi can be implemented to make transfers between devices considering the files generated by the datalogger. Another component incorporated to the devices developed during this research work is the Global System for Mobile Communications (GSM) as reported in [17]. However, using such a service requires payment of a fee, which represents one of its limitations. For instance, in this case, data must be reloaded, and a cell phone number must be assigned to every node.

The study of acceleration or vibration is another relevant method to compute the dynamic performance of structures [6,18,19]. However, most of the research on vibration extraction is developed with the help of acceleration devices or vibrometers. In most of the cases, such devices are utilized for industrial applications which may be an expensive method. Thus, the use of expensive vibrometers is exclusive for a sector of society that can afford it. As an alternative to the above-mentioned device, there are other sensors, perhaps less sensitive but accurate enough to achieve a solid resolution that could be used in SHM philosophy. An example of these sensors are the Micro Electronic Mechanical System (MEMS) accelerometers. MEMSes are composed by microscopic elements that can fold, expand, move, and basically perform any type of movement in a microscopic environment; hence, accelerometers that use this technology are generally built with a “spring mass” system where a metal plate moves between two points generating energy every time the plates touch each other. To determine the acceleration, these accelerometers use the time it takes for the mass to move. These electronic devices are microscopic, and they are extremely sensitive to movements that can be recorded with a pinpoint accuracy [20]. Some studies have reported that MEMS accelerometers are suitable for monitoring in cases where the acceleration rate is used to detect fatigue and structural failures [21,22,23,24]. Even in [25], the design, fabrication, simulation, and measurement of a single-axis piezoelectric MEMS accelerometer sensor based on the aerosol deposition of a layer of piezoelectric material (PZT) is presented. In addition, there are some other technical studies reporting the calculation of the quasi-static behavior of structures by the integration of vibration and inclination data [26]. The above-mentioned publications endorse the importance of MEMS accelerometers for SHM techniques. In the case of this paper, a novel acceleration sensor is developed to be implemented in the SHM of structures. Within this context, a MPU9250 sensor was chosen, since it had already been validated in [27], by making a comparison through monitoring under equal conditions with a piezotronics 352C03 [28].

Since the dynamic response of structures propagates in the form of waves, it is important to measure such a propagation to detect how these waves are distributed along the structure under consideration. The extracted data coming from structural wave monitoring can be subsequently post processed to obtain important structural health data. These data can be obtained by implementing mathematical methods such as polynomial regression or modern approaches as machine learning [29]. The latter can be used to detect whether external factors such as the change in speed of a railway causing tension and considerable deformations on the tracks. In general, if the propagation of waves is known, it may be possible to compute the tension and/or torsion being applied to the tracks, and therefore their deformations [30]. In this regard, it is necessary to measure and establish the propagation of vibration of structures to prevent failures. However, the synchronization of all the nodes in the system employed to carry out the monitoring process at the beginning of the measurement is fundamental to achieve the above-mentioned computations. If this last condition is not met, the travel time of vibration waves is lost, and the monitoring process must be restarted. As a result of this last situation, different synchronization techniques have been proposed to implement when obtaining sound measurements. Examples of such techniques are the Reference broadcast Synchronization (RBS) [31], Time Sync Protocol for sensor Network (TPSN) [32], Flooding Time Synchronization Protocol (FTSP) [33], Pulse Per Second Sync (PPS) [34,35], Flooding with Clock Speed agreement (FCSA) [36], gradient time synchronization protocol (GTSP) [37], etc. Based on the study documented in [38], the existence of an error between pulses of approximately ±400 ns was found; however, it turns out to be the best synchronization method, since the wireless system can be easily implemented on it.

In summary, a monitoring system was developed in this research using a GPS receiver to extract the PPS, and a synchronization approach is integrated between wireless, low power consumption, and long-range monitoring nodes. The implementation of the method was validated with the help of an experiment through which the propagation of vibrations on a steel I-beam was obtained. Within this context, the fundamental frequency of the beam was extracted as well. Based on the results, it was more than clear than this method represents a viable contribution to the SHM philosophy.

## 2. Materials and Methods

To carry out the monitoring of vibration propagation, it was imperative to develop a system to detect mechanical vibrations. Within this frame of reference, the research team decided to synchronize wireless nodes through the RTC+PPS method. The datalogger utilized in the proposed system is centered around the System on a Chip (SoC) ESP WROOM 32 by Espressif Systems, hereafter referred as simply ESP32. The extracted measurements are executed at a rate of 100 samples per second, utilizing Invensense MP9250, which is a 3-axis MEMS accelerometer. This accelerometer also integrates a 3-axis gyroscope and magnetometer. Furthermore, to connect the MPU9250 acceleration sensor to ESP32 SoCs, four category 5 UTP cables were used. To ensure that the SPI communication, operating at a transmission frequency of 4 MHz, does not experience data loss during the transfer through the cables, their length was set to fifty centimeters. The acquired data were stored in the ESP32’s RAM memory, which had a capacity of 520 KB for sketches. These data sets were transmitted via the RFM69 module, a long-range radio operating system working at a frequency of 915 MHz. Table 1 describes in detail each of the components from the monitoring node.

**Figure 1:**
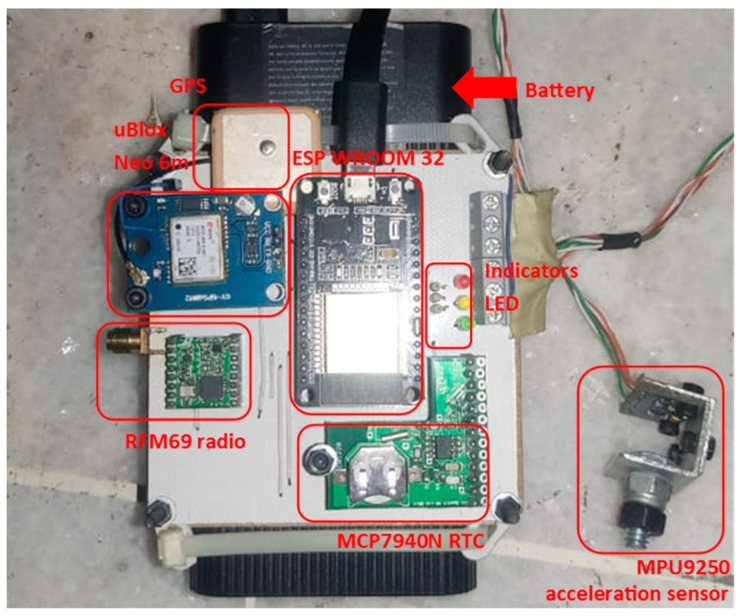
Monitoring node and its components.

Figure 1 illustrates an example of the PCB (Printed Circuit Board) card of one of the nodes where the sensor, the RTC, the GPS, and the SoC are connected. In this experiment, two of these nodes were used to perform the monitoring synchronization; these nodes are called monitoring nodes or client nodes within the RTC+PPS synchronization system.

The monitoring and receiving node, respectively, are equipped with a synchronization method based on an MCP7940 Real-Time Clock (RTC) and a Global Positioning System (GPS) providing a PPS signal. The method involves the use of an RTC to generate a “trigger time” which is sent from the receiving node (now referred to as the master node) to all monitoring nodes. After receiving the trigger time from the master node, the monitoring nodes wait 10 s and then anticipate a PPS signal. These nodes receive this PPS signal at the same time, thereby guaranteeing the synchronization required between nodes.

**Figure 2:**
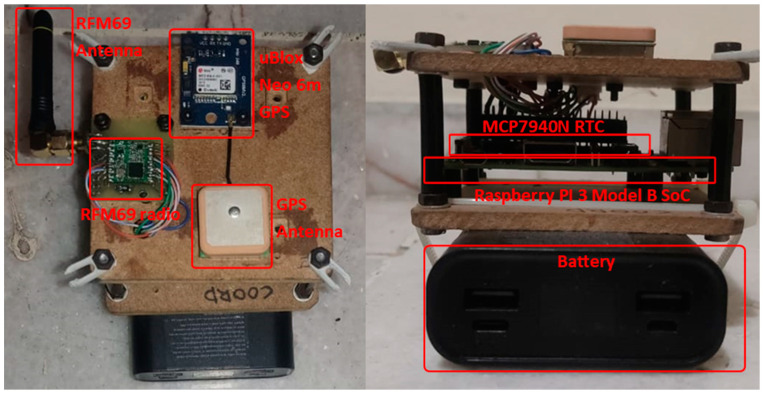
Master node and its components.

The system also encompasses a receiving node where data from the monitoring nodes are stored in a simple comma-separated value (.csv) text file. At its core, this node can be seen in Figure 2 and employs a Raspberry Pi 3 Model B SoC. It is equipped with a 64 GB micro-SD memory card for local storage, featuring 2.4 GHz wireless communication compliant with IEEE 802.11 b/g/n and Bluetooth 4.1. Additionally, it includes 40 general-purpose input/output (GPIO) pins. Table 2 describes in detail each of the components of the master node.

**Figure 3:**
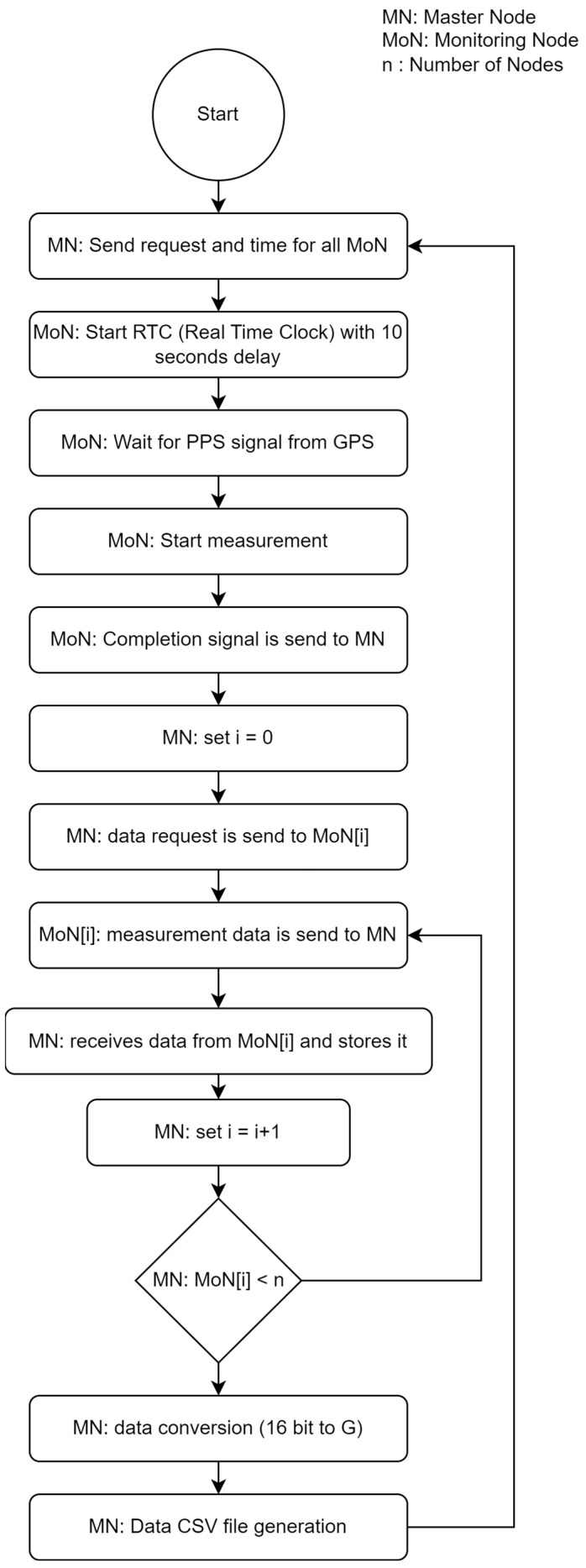
Flowchart of the RTC+PPS monitoring synchronization system.

The algorithm to perform the monitoring process is designed within the Arduino environment which is compatible with ESP32 SoC boards where only “Wire.h”, “SPI.h”, and “RFM69.h” were utilized as libraries for the end node firmware development. Each of the tasks for performing measurements is separated into functions. The system operates as follows: the master node sends a trigger signal containing the exact time when the measurement was recorded. This signal is compared by the monitoring nodes, and when the specified 10 s time interval elapses, monitoring begins for 30 s. This method is called RTC+PPS monitoring synchronization system. Once the monitoring process is completed, the monitoring nodes enter on a standby mode. The master node sends a request signal to initiate data reception from the monitoring nodes, starting with Node i. Each node starts transmitting data in batches of 60 bytes. Upon completing the transmission, each node sends a termination signal. As the master node receives data from Node i, it stores the data in RAM and proceeds to request data from Node i + 1. The process continues, with the master node sequentially receiving from all nodes and storing data in RAM. Subsequently, the master node combines lower and higher bytes, forming 16-bit strings for each measurement. Finally, all the data are stored in local memory for subsequent processing. This process is described in detail in Figure 3.

## 3. Validation

### 3.1. Accelerometer

**Figure 4:**
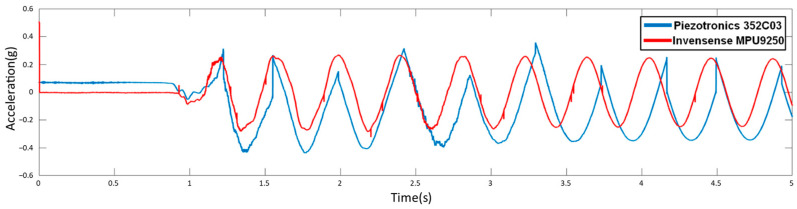
Comparison data between acceleration sensors through sine wave excitation [27].

In the M.S. thesis of the first author, the MPU9250 sensor was validated through a comparison with the help of a Piezotronics 352C03 sensor; more information can be found in [27]. In summary, for validation, the sensors were positioned at the same distance on a steel beam with a type I cross-section. The beam was excited by a shake at one of its ends, and its spectrum was obtained implementing a Fast Fourier Transformation (FFT). Figure 4 presents the obtained response of the beam considering the above-mentioned sensors. In general, in Figure 4, it can be observed the response of the beam being monitored by both sensors (MPU9250 and Piezotronics 352C03). It is interesting to observe that both synchronized signals overlay very well with each other. A very similar response and tendency is detected. The reason why the red signal exhibits a larger wave is due to its resolution being configured at ±16 g, while the blue signal is set at ±8 g. Nonetheless, both signals exhibit the same behavior at their respective times.

**Figure 5:**
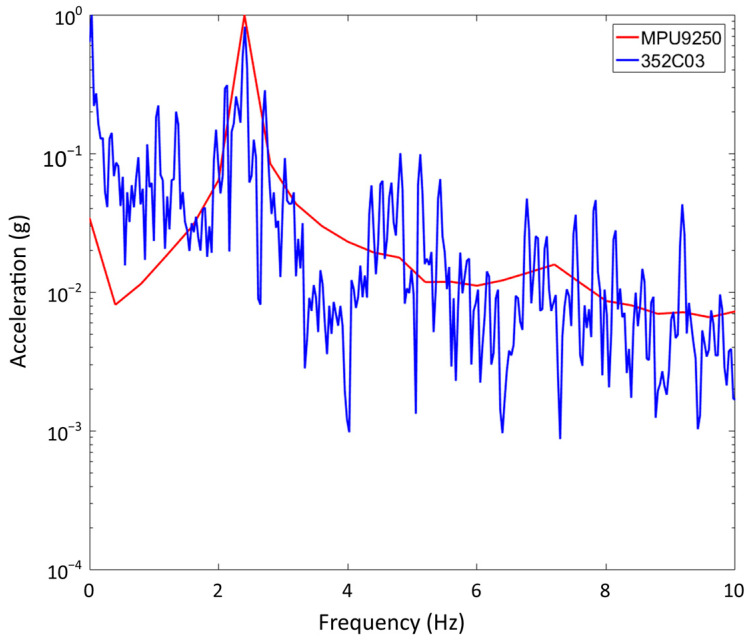
Comparison of MPU9250 and 352C03 sensors in the frequency domain [27].

Figure 5 illustrates the FFT obtained considering the MPU9250 and 352C03 sensors, respectively. In other words, Figure 5 illustrates the response spectrum of both sensors. the frequencies of both signals are observed as well; the signal in red corresponds to the MPU9250 sensor and the signal in blue to the 352C03 sensor. In addition, in both cases, the highest responses are located at the same frequency to that of the fundamental frequency of the beam under study. Based on these results, it can be established that the Invensense MPU9250 and the piezotronics 352C03 sensor extract very similar responses in the frequency domain. In general, both sensors have the same capabilities to extract the fundamental frequency of the beam; therefore, it is evident that the Invensense MPU9250 sensor is suitable for the extraction of structural data to calculate dynamic characteristics of structures using vibration waves.

### 3.2. Signal RTC+PPS

One of the greatest challenges for the signal RTC+PPS experiment was to achieve the initial synchronization for the monitoring nodes. The way they were initially programmed did not account for this synchronization from the outset. Despite this, monitoring was being conducted in laboratory tests, resulting in data from both sensors. However, it was not possible to determine which node was initiating the measurement. Thus, to identify signal propagation, synchronization of the data was necessary.

To address this issue, the synchronization method proposed in [34] was employed. This method involves using the PPS signal provided by a GPS and utilizing it as a trigger signal for data synchronization. The method is quite straightforward. From the Ublox Neo 6M GPS, a PPS signal output is available—a pulse with a frequency of 1 s. This signal is satellite-synchronized, resulting in precise timing accuracy. However, according to [34], this method is not sufficient for achieving synchronization in all measurements. In this sense, there are instances of exact synchronization and others that are not synchronized due to the nature of microcontrollers. Then, they need to start at the exact same time from the beginning. To address this, another long-standing technology for synchronization, the real-time clock, was implemented.

**Figure 6:**
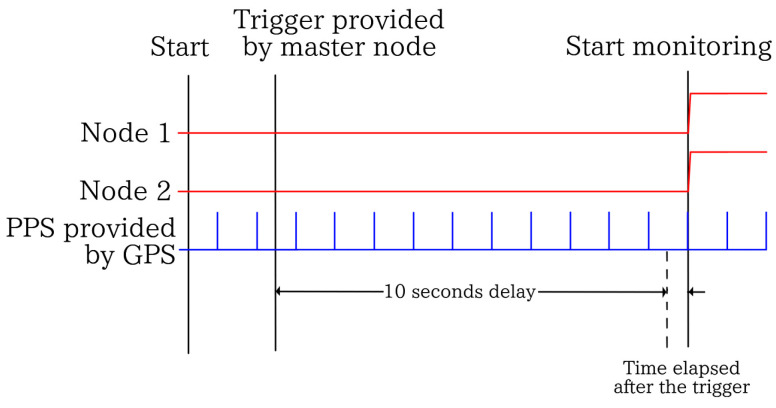
Execution time of the GPS trigger-based monitoring.

The main issue of data desynchronization arises because, at times, the triggering of monitoring experiences a time shift of around ±1 s. This results in imprecise synchronization. Consequently, an RTC controller was installed to address this problem. By controlling the start time of monitoring, it becomes possible to regulate each monitoring instance. The algorithm operates as follows. Initially, the exact time is unknown for each sensor. In contrast, for the master node, it is merely a matter of requesting the system time. Subsequently, the master node transmits the exact time via radio to all monitoring nodes simultaneously. Now, for each node within the system, based on the registered time, an alarm is set ten seconds after the time provided by the master node. This mitigates the time discrepancy, as if the request falls within the interval of a second, it is impossible that the PPS signal will be encountered at the end of the countdown. In such cases, the node is simply put into a standby mode, activating the monitoring function upon receiving the PPS signal, this is illustrated in Figure 6.

With the help of the above-mentioned approach, it is possible to guarantee that each of the measurements will be synchronized, eliminating the need to record unsynchronized monitoring measurements. Nevertheless, since the entire system relies on a GPS receiver module, it is influenced by environmental conditions. Therefore, it is not recommended to conduct monitoring under cloudy skies or near structures that might cause GPS signal loss.

**Figure 7:**
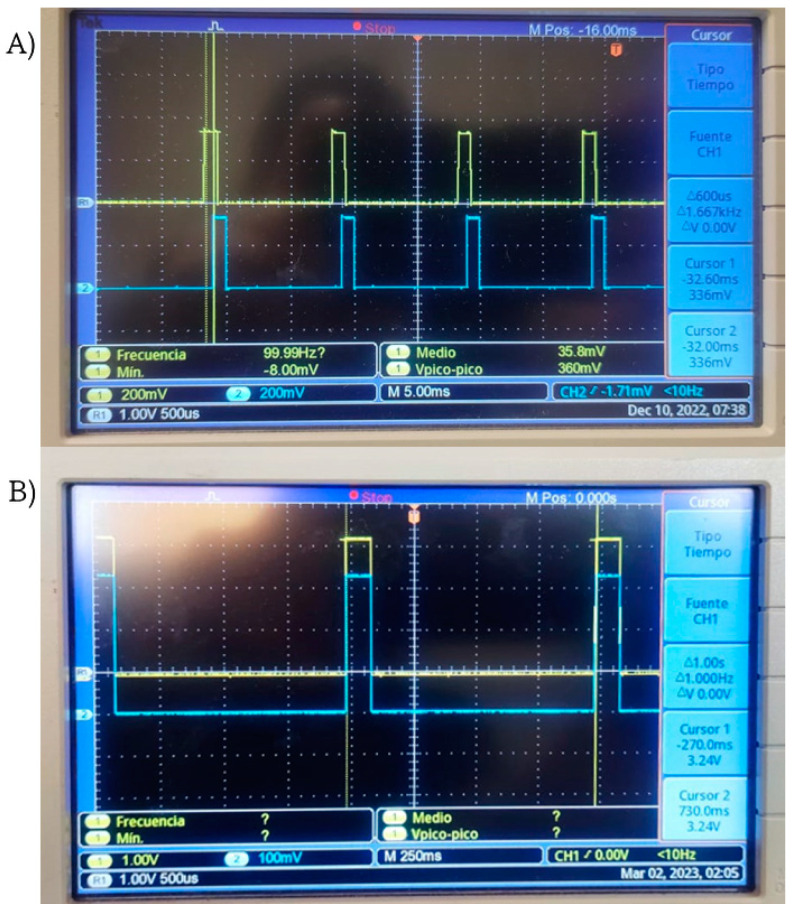
Comparison between unsynchronized system (**A**) and synchronized system using the RTC+PPS method (**B**).

With the aim to validate the accuracy of the presented approach, a laboratory test was conducted where, for each measurement, a 20 μs pulse was sent. Each sensor was connected to an oscilloscope terminal, and monitoring was performed. In Figure 7A, desynchronized data can be observed, whereas in Figure 7B, synchronized data using the RTC+PPS method are displayed.

### 3.3. Radio RFM69

To validate the RFM69 radio module, a low-power prototype based on two radios was designed and constructed. Radio 1 was configured as a data transmitter; then, data were sent which is originated from the reading of a variable resistor. On the other hand, Radio 2 was set in receiver mode and continuously received real-time data.

**Figure 8:**
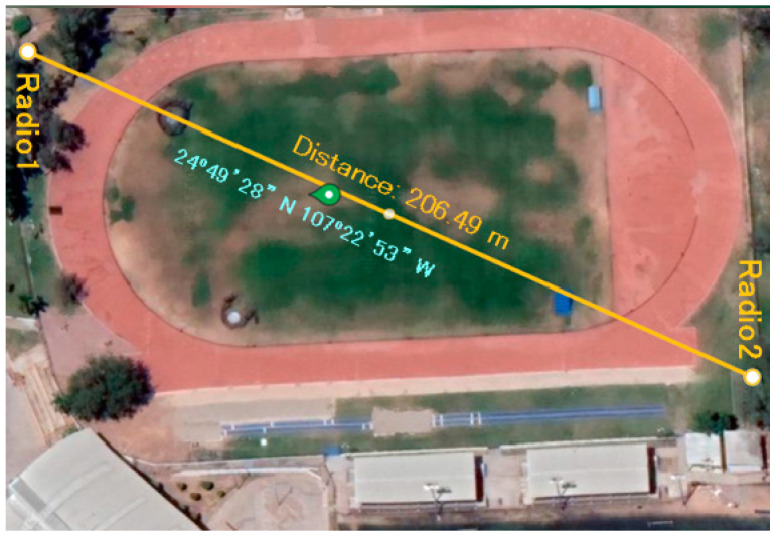
Distance between two RFM69 radios on the campus running track 24°49′28″ N 107°22′53″ W of the Autonomous University of Sinaloa in Culiacan, Mexico.

The experiment illustrated in Figure 8 aimed to test the signal loss between Radio 1 and 2. To determine if the signal was lost, a 128 × 64 OLED was implemented to display the received data. Hence, if the signal was lost, the display showed “NO SIGNAL”. However, despite increasing the distance between the sensors, the connection remained uninterrupted. This result was obtained in a scenario without buildings and where the signal path between the radios was always in a straight line. The experiment was conducted on the running track in the main campus of the Autonomous University of Sinaloa in Mexico. The location with the longest available distance did meet the mentioned criteria. The maximum distance achieved was approximately 206 m, as represented in Figure 8. Based on this last fact, an important observation can be stated as follows. Since bridges where these devices were planned to be implemented have spans of less than 200 m approximately, it is feasible the use of them to monitor the structural behavior of those structures.

### 3.4. Data Integrity

One issue encountered during data transmission is occasional data corruption, where some of the data are not transmitted correctly. To address this problem, it is necessary to activate the ACK response function. Next, an example of how it works in a point-to-point radio system is introduced.

In a system composed of two radios, with Radio 1 configured as the transmitter and Radio 2 as the receiver, Radio 1 sends a message to Radio 2. Upon receiving the message correctly, Radio 2 sends an ACK message back to Radio 1. If Radio 1 receives this ACK message, it acknowledges that the transmission was successful and completed. However, if ACK is not received by Radio 1 within a 100 ms period, it cancels the entire transmission and displays an error message on the screen. Radio 1 remains in a waiting mode until a user presses the RESET button on the SoC ESP32. This mechanism helps ensure data integrity and prompt error detection during the transmission process.

This function is incorporated into the monitoring system; specifically, this condition is active in all nodes to ensure that no nonexistent data are present in transmissions. At this point, the entire chain of acceleration data can be expected to reach from each node to the master node. Therefore, the method used to send the data is as follows:

Upon initiating the monitoring process, all data corresponding to a 30 s measurement are stored in a character string. It is important to note that the MPU9250 sensor has a resolution of 16 bits for each axis of acceleration. Now, the SPI port only allows transmission with each message composed of 8 bits. As a result, the complete message is divided into two parts: the Most Significant Bits (MSB) and the Least Significant Bits (LSB). The MSB contains the most significant bits of the data, and the LSB contains the least significant bits. It is crucial to transmit this message correctly to prevent data corruption.

The structure of a six-message, 8-bit packet is as follows: MSB_X, LSB_X, MSB_Y, LSB_Y, MSB_Z, and LSB_Z, respectively. This batch of 6 bytes is sent in a single 60-byte chain. It is then complemented with the next nine measurements and sent in batches of 60 characters, one by one, until the entire chain is transmitted. With knowledge of the chain’s structure, the message can be decoded, and all acceleration data can be restructured in the master node. This method ensures the proper and organized transmission and reception of data from each node to the master node.

To correctly decode the message, it is important to understand that the MPU9250 delivers data in binary format using the complement of two. This means that the most significant bit corresponds to the sign of the number, which can be negative or positive. As it is a 16-bit chain, the sensor provides data ranging from −32,768 to 32,767.

With this understanding, the data can be easily transformed from raw to acceleration by performing a simple division. With the help of the sensitivity setting of the sensor, in the case of ±16 g, the desired real-world value in signed decimal format (x) value must be derived as follows:

(1) $$x=\frac{dv}{32767}\times S,$$

where *dv* is the digital value in binary two’s complement; *S* is the sensitivity of sensor in this case equal to 16.

## 4. Full-Scale Experiment

**Figure 9:**
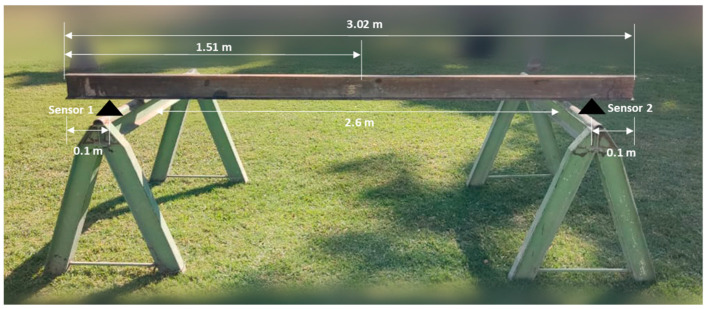
Full-scale synchronization experiment.

The experimental part of this paper consisted of the structural monitoring of a steel beam with a type I cross-section. The beam was simply supported in two points along its length. The distance between the two supports was 2.6 m, and beyond the two supports in both sides the beam was a cantilever part with a length of 0.1 m. Figure 9 illustrates the configuration of the experiment.

The MPU9250 sensors were placed on the underside of the beam, 10 cm from each end as illustrated in Figure 9. To determine the natural frequency, measurements were initially taken at a 100 Hz sampling rate due to hardware limitations. However, the latest update of the monitoring nodes resolved these limitations. Nonetheless, the measurements were still conducted at 100 Hz as the code had already been configured for that sampling frequency.

For each monitoring conducted in this experiment, the beam was excited at one of its ends to induce vibration in the structure, aiming to determine its fundamental frequency. To excite the beam, a 200 g rubber mallet hammer is used to strike its end. The strike should not be too forceful or too gentle. Through experimentation, it was determined that the ideal strike is simply allowing the mallet fall under its own weight to hit the structure. This ensures a moderate impact that is not overly powerful, yet not too feeble, considering the low frequency of impact. Additionally, striking at the same point on the beam is crucial. To achieve this, a target is placed at the designated point.

During the experiment, data were collected from 30 impacts on each side of the beam under consistent conditions between 16:00 and 19:00 h of the day. This time frame was chosen to minimize the impact of temperature on the structure, given that temperatures in Culiacan, Mexico may reach up to 40 degrees Celsius. High temperatures could lead to metal expansion, potentially causing variations in recorded frequencies during measurements.

## 5. Results

The objective of this study was to evaluate the performance of a wireless vibration monitoring system implemented on a steel beam with a type I cross-section. The system employed synchronized wireless nodes equipped with MPU9250 accelerometers, utilizing the RTC+PPS synchronization method. A series of impact tests were conducted on the beam’s end using a 200 g hammer, resulting in 30 monitored instances. The aim was to assess the system’s ability to accurately capture and analyze structural vibrations.

### 5.1. Synchronization Success

**Figure 10:**
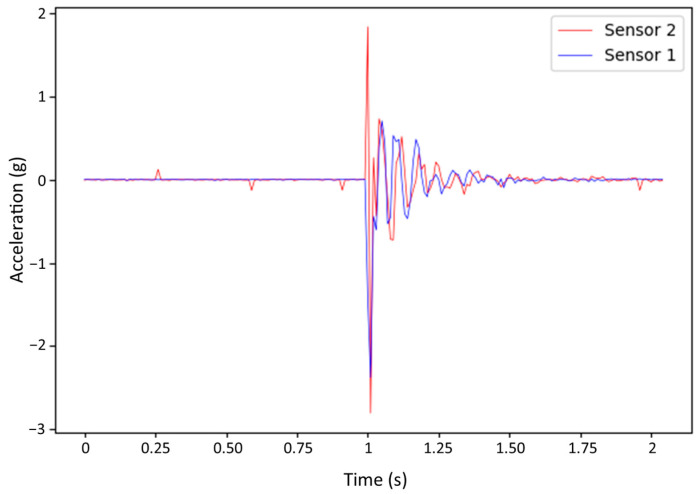
Example of synchronized monitoring session for both nodes with Impact at End A of the beam.

The experiment began by assessing the effectiveness of the RTC+PPS synchronization method. The results indicated a high degree of success, as data acquisition across all monitored nodes was precisely coordinated. The synchronized data demonstrated minimal time deviation, ensuring accurate timing alignment among the nodes. This synchronization method was vital in establishing a consistent baseline for subsequent analyses. Figure 10 shows the signal belonging to one of the synchronized measurements in the experiment.

### 5.2. Propagation Analysis

**Figure 11:**
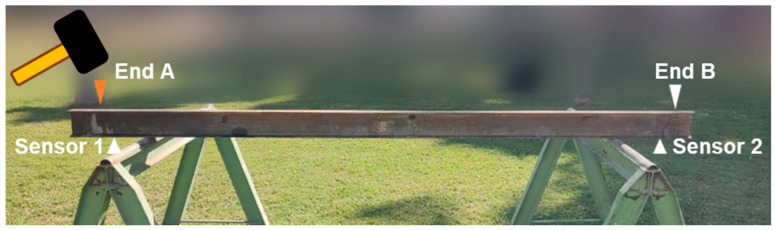
Impact on End A of the beam.

Propagation analysis was carried out by performing 30 acquisition tests of 1024 samples each that were organized as four statistical groups labeled as RTC+PPS sensor 1, RTC+PPS sensor 2, Unsynchronized sensor 1 and Unsynchronized sensor 2. In addition, these tests were performed twice, one for the case where the hammer impact comes from Point A and a second group of 120 tests for the four groups where the impact comes from Point B. As an example, Figure 11 shows the case where the hammer impact comes from the A point of the beam. Based on this, the behavior of the structure as well as its natural frequency can be extracted. As specified earlier, the impact is carried out using a 200 g hammer. For each monitoring, only one impact is performed, so each statistical group requires a total of 30 monitoring tests. Furthermore, a comparison is made between synchronized and unsynchronized data.

In summary, Figure 11 provides an illustrative example of an impact on the beam. Prior to delving into behavior analysis, it is essential to address certain signal intricacies. Primarily, both signals initiate simultaneously, suggesting an absence of apparent propagation, at least in this monitoring instance. Consequently, a closer scrutiny of the signal is requisite to accurately interpret the results.

**Figure 12:**
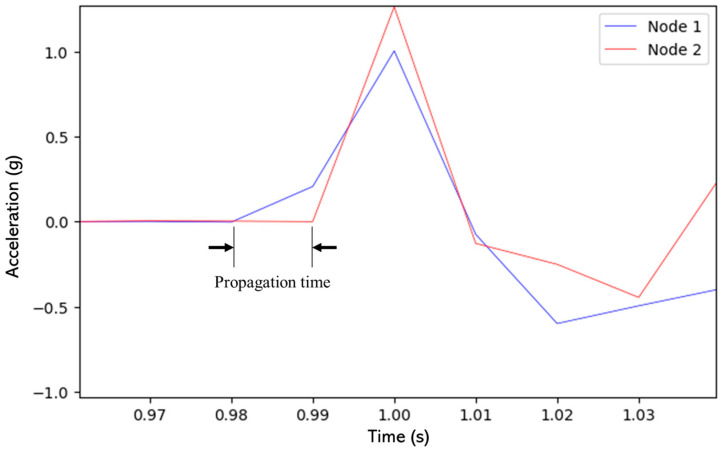
Propagation time of Impact at End A of the I-Beam where the Node 1 is a blue signal and Node 2 is a red signal.

As depicted in Figure 12, it is only upon closer inspection that propagation time can be found, which is defined as the time it takes for the signal to arrive from Point A to Point B on the I-beam. Since both sensors start monitoring at the same time, propagation time can be understood as the phase delay between the start of the vibration behavior in the acceleration signals from both nodes. The blue signal corresponds to Sensor 1 signal while the red signal represents Sensor 2 signal.

Deeper analysis involving signal processing techniques is essential to unveil any potential propagation trends that might be present in the data. These insights could hold crucial information about the beam’s dynamic response to impact.

### 5.3. Spectrum Analysis

**Figure 13:**
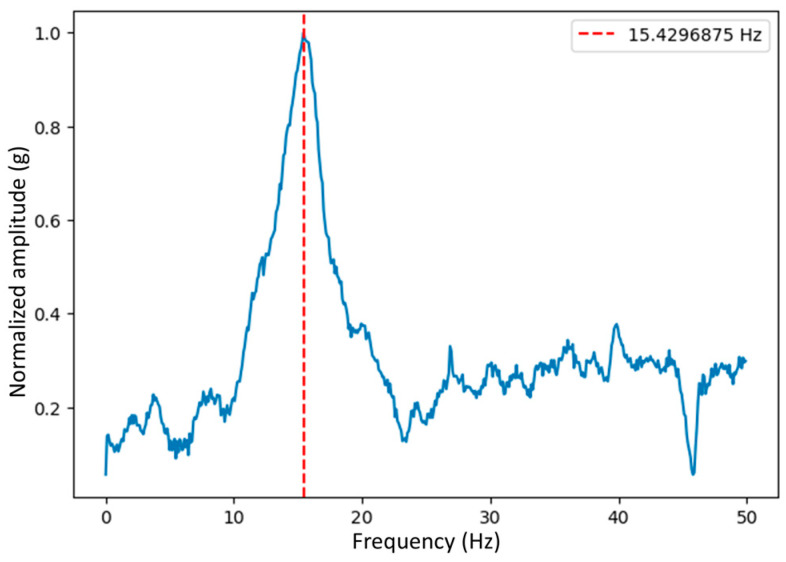
Monitoring of fundamental frequency of the I-beam, impact on A.

Figure 13 shows the spectrum of one of the results and a graph of these, omitting the atypical values of the tests, demonstrating that the fundamental frequency of the I-beam can be obtained by this method.

To determine the fundamental frequency of the beam, an analysis of the spectrum was also carried out for each monitoring. After all the frequencies were extracted considering every monitoring process, they were plotted to validate if the method can report the behavior of the beam as well as present gradual changes in the system. Hence, if the fundamental frequencies vary over time, it is an indicator that the beam is presenting changes in its composition, which could be translated as structural deterioration.

Table 3 and Table 4 summarize the results of the fundamental frequency determination by sensors with respect to impacts on Points A and B, respectively. Based on the statistical results, differences between synchronized and unsynchronized devices can be observed after performing 30 epochs for each group. In addition, a more stable behavior in the RTC-PPS synchronized groups can be noted compared to non-synchronized monitoring tests. Sometimes it is possible to determine the fundamental frequency, as in the case of Sensor 2, but other times not, as in the case of Sensor 1, where it can be seen how it is a set that is too dispersed and far from the average, without mentioning that this set is the only one that could not find the fundamental frequency that is close to 15.5 Hz. In summary, it can be established that RTC-PPS tests present lower standard deviations and overall better statistical performance, demonstrating that synchronized devices are more accurate to determine fundamental frequency.

**Figure 14:**
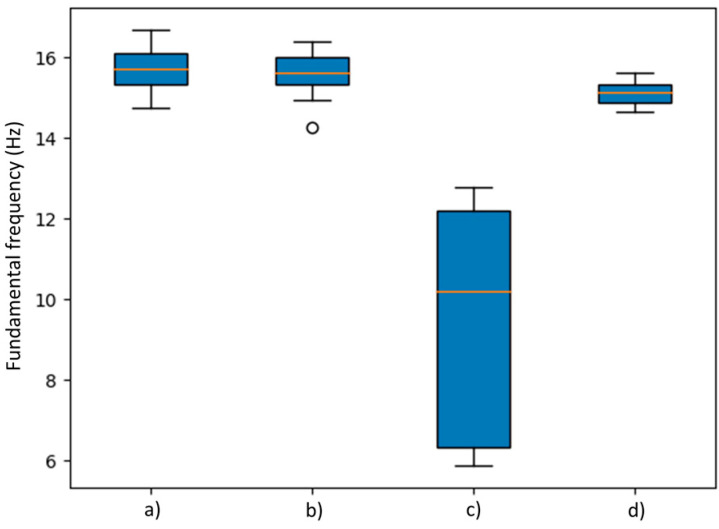
Box plot analysis of fundamental frequency estimation by statistical group with impact at end A of the I-beam where (a) is RTC+PPS sensor 1, (b) is RTC+PPS sensor 2, (c) is Unsynchronized sensor 1 and (d) is Unsynchronized sensor 2.

It is interesting to mention that the response of the system is different when the monitoring is not synchronized because, as it can be seen in Figure 14, when the system is not correctly synchronized, this imbalance causes one of its sensors to not report the fundamental frequency correctly. 

Figure 14 illustrates the comparison between data obtained by the RTC+PPS synchronization method versus the non-synchronized method of fundamental frequency data from measurements of the I-beam when the impact occurs at End A. These data are distributed for each method into two sets of measurements, corresponding to sensors one and two, respectively. These boxplots illustrate the dataset’s behavior, with the median represented by a red line. The blue-shaded areas above and below the average line represent the upper and lower quartiles, while the outer graduations represent the upper and lower extremes. Lastly, points beyond the limits of the bars indicate outliers, resulting from corruptions in the data presented on the monitoring due to various factors such as signal transmission noise pollution or uncontrolled impacts on the I-beam. When the impact is received at End A of the beam, it is observed that when the vibration data are taken with the RTC+PPS synchronization method the group of frequencies is stable, while when they are not synchronized, the vibration data present data sets that are distributed in a non-uniform way.

**Figure 15:**
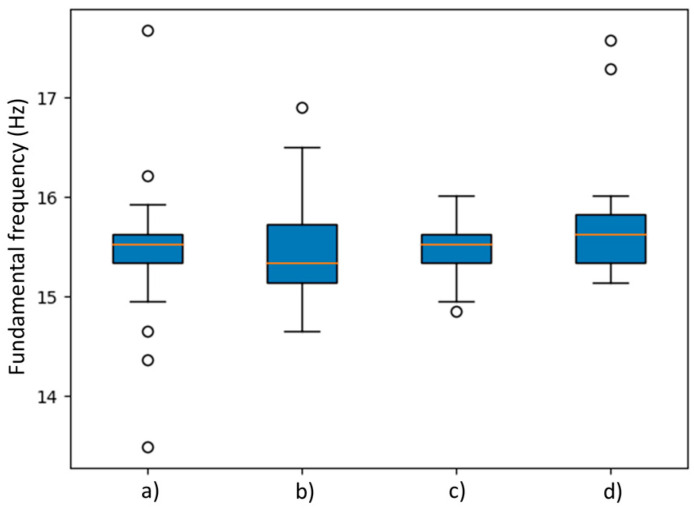
Box plot analysis of fundamental frequency estimation by statistical group with impact at end B of the I-beam where (a) is RTC+PPS sensor 1, (b) is RTC+PPS sensor 2, (c) is Unsynchronized sensor 1 and (d) is Unsynchronized sensor 2.

In the case of monitoring the impacts at End B of the beam as shown in Figure 15, all the data sets are stable.

## 6. Discussion

As it is observed in Figure 10, results demonstrate that this wireless monitoring system can acquire vibration signals from two sensors in a proper way, as many authors have previously reported. However, this study focuses on proposing a new synchronization methodology to activate two or more wireless vibration sensors, and at the same time, measure vibration propagation in structures. Because of this, it is very important to generate a synchronized trigger signal to activate end nodes at the same time. In addition, it is important to mention that common wireless sensor networks start measurement processes by sending activation commands to end nodes one by one in a sequential way. This is similar as the unsynchronized tests statistical groups reported in this project. On the one hand, sequential activation commands make difficult to measure vibration propagation in structures because of the time elapsed between end nodes activation commands as illustrated in Figure 7. Furthermore, the proposed methodology relies on a trigger strategy that consist of sending a common date–time start value in a sequential way to all end nodes to establish a common RTC-based trigger. However, activation times between end nodes provokes the loss of data associated to vibration propagation, because propagation time could be close to sequential activation times. This is where this project proposes to utilize the PPS signal from Ublox Neo 6M modules as acquisition trigger once the previously preprogrammed common start datetime is reached by all end node RTC+PPS devices.

In addition, the propagation analysis experiment was carried out by acquiring 30 tests for each statistical group and these groups were organized as Sensor 1 unsynchronized, Sensor 2 unsynchronized, Sensor 1 RTC+PPS and Sensor 2 RTC+PPS. Consequently, synchronization results demonstrate that the RTC+PPS synchronization method is reliable and reduces the error by reducing the elapsed time between activation commands in RTC+PPS based devices as it can be seen in Figure 7, ensuring that a given time between Sensor 1 and 2 vibration signals should be due to propagation time as it occurs in Figure 12 signals instead of measurement errors due to activation delay between end nodes. Thus, the exact time in which a vibration signal travels from Point A to Point B in a mechanical structure can be detected. However, due to the utilized sampling frequency (100 Hz), propagation time can be measured with 10 ms time resolution.

To validate the effectiveness of this methodology, fundamental frequency estimation of a beam structure was chosen as parameter of study by comparing fundamental frequency determination on the four statistical groups. Statistical analyzes were carried out by calculating mean, standard deviation (SD) and variation coefficient (CV) from the fundamental frequency determination result from each vibration measurement and arranging the data according to the previously mentioned statistical groups to compare their statistical properties. In Figure 13, it can be observed an example of a vibration spectrum with its fundamental frequency determination on the A side of the beam.

As summarized in Table 3, results indicate that the mean of fundamental frequency estimation of Unsynchronized sensor 1 is far from the other groups value while SD and CV parameters result higher for this group indicating that fundamental frequency estimation is not reliable for Sensor 1 at Point A of the beam when unsynchronized trigger is utilized. Counter-wise, Table 3 shows that RTC+PPS synchronized devices and Unsynchronized sensor 1 have a fundamental frequency of around 15.5 Hz, which is similar between them, and small SD and CV values, which indicates a reliable fundamental frequency estimation on the A side of the beam.

Additionally, the results summarized in Table 4 present a similar behavior between statistical groups, as observed in Table 3. However, in this case, results of the B point show that Unsynchronized sensor 2 tests obtain a fundamental frequency mean of 16.178 Hz which is slightly different from the rest of the statistical groups that obtain a value of around 15.4 Hz. In addition, SD and CV parameters are higher on Unsynchronized sensor 2 test group when it is compared from the other statistical groups. Because of this, it can be observed that RTC+PPS-based tests provide a more reliable fundamental frequency estimation than unsynchronized devices due to precise synchronized trigger signal. It is important to mention that the most unreliable statistical groups were the ones that utilized unsynchronized trigger and the sensors located directly below the impact point of each scenario. It should be noted that this phenomenon does not occur in cases where the accelerometer is not directly below the point of impact. In these cases, the coefficient of variation is very close to 1.68%. On the contrary, when the RTC+PPS synchronization method is applied, this phenomenon does not occur. 

Finally, box plot analyses were carried out on Figure 14 and Figure 15, where it is seen that orange line represents the median of each statistical group. On one side, it can be observed that Figure 14 (c) box has a wider length due to a high variation between estimated fundamental frequency values and a median value that is located far from the (a), (b) and (d) groups which come out to be like Table 3 results. On the other side, Figure 15 shows the results where box plots are similar between groups where median values are around 15.5 Hz. This can be understood as better performance on End B tests. However, some atypical values can be observed in Figure 15 as small circles outside of the whiskers of each box.

## 7. Conclusions

In this research, the development of a whole wireless accelerometer sensors network system was proposed for vibration propagation measurement. As it was previously mentioned, conventional wireless sensor networks utilize sequential measurement activation when multiple sensor reading is required. Consequently, a delay time appears between end node to end node measurement activation signals. In addition, vibration propagation is the phenomenon where mechanical vibrations travel across a solid structure and propagation time is the time that takes for a stimulus to travel from a starting point to other point in a structure. However, propagation time is often small in rigid structures and could be difficult to be measured when sequential activation is utilized to measure multiple end nodes in a sensor network. This is where synchronization is required to activate multiple sensors reading at the same time, and RTC+PPS synchronized methodology was compared to a conventional sequential reading in this project. The overall statistical results showed that RTC+PPS synchronized end nodes provided a better and more reliable performance compared to its unsynchronized counterparts that just relied on RTC as synchronization signal source. 

In this research, the results show that the developed RTC+PPS-based synchronization system is reliable for the detection of vibration propagations in a structure. Because of this, it is possible to obtain a precise synchronization between monitoring nodes and the master node. An experimental validation of the system was carried out. In addition, the wireless communication between the nodes was evaluated using RFM69 radios. A distance test was carried out where the nodes managed to maintain the connection at more than 200 m, demonstrating its viability for bridge monitoring applications. To ensure the integrity of the data, the transfer confirmation function that validates the successful reception of the data transmitted between nodes was implemented. This helped to minimize data loss and ensured the reliability of the measurements.

Full-scale experimentation on an industrial steel beam was carried out and provided valuable information on the vibration behavior of the structure. The obtained data made it possible to identify the fundamental frequency of the beam and to analyze the propagation of vibrations along its length. The precise synchronization and transfer confirmation feature ensured that the data were reliable and consistent, which is essential for accurate interpretation of results and decision making for SHM.

Furthermore, it can be established that the proposed methodology was effective to determine the fundamental frequency with a value around 15.5 Hz in most of the cases according to the Table 3 and Table 4, Figure 14 and Figure 15 results. However, an advantage on the RTC+PPS synchronized devices can be observed due to its precise trigger methodology that allows reduction in SD and CV in the tests, making this methodology more reliable for vibration monitoring when multiple sensors reading is required.

In conclusion, this wireless monitoring system can be utilized for the acquisition of useful synchronized vibration data on multiple points of bridges and other structures with the advantage of a wireless synchronization trigger that does not require wiring the structure for synchronization nor for data transmission. In addition, it is important to mention that this methodology relies on GPS atomic clock to provide an almost perfect synchronized PPS signal to be utilized as measurement trigger source. Finally, it can be concluded that the combination of precise synchronized timing, reliable and long-range communication makes it a valuable tool for early detection of structural problems and improved management and maintenance of critical infrastructure.

## Figures and Tables

**Table 1:** Characteristics comprising the body of a monitoring node.

Component	Model	Technical Characteristic	Functionality
SoC	Espressif ESP WROOM 32, Espressif Systems, China	CPU: Dual core Tensilica Xtensa LX6 (32 bit).	It is the primary System on a Chip (SoC) responsible for running the monitoring program.
		Clock frequency: 240 MHz.	
		Voltage: 3.3 V DC, 80~500 mA.	
		40 GPIO.	
		Memory: 4 MB Flash, 520 KB RAM.	
		Wireless: 2.4 GHz IEEE 802.11 b/g/n and Bluetooth 4.1.	
Accelerometer Module	InvenSense MPU9250	Triple-axis MEMS accelerometers.	It is the triaxial acceleration sensor, responsible for conducting monitoring at a sampling frequency of 100 Hz.
		Scale programmable ±2 g, ±4 g-force, ±8 g and ±16 g.	
		16 bits ADC.	
		Normal current operation: 450 µA.	
		Low accelerometer mode current: 8.4 µA at 0.98 Hz, 19.8 µA at 31.25 Hz.	
		Sleep mode current: 8 µA.	
GPS module	u-blox Neo 6M	Horizontal Position Accuracy: ±2.5 m	It is the GPS receiver that provides the PPS signal used for synchronizing the monitoring system.
		Communication Protocol: NMEA, UBX Binary, RTCM	
		Navigation Sensitivity: −161 dBm.	
		Operating Voltage. Provides PPS signal.	
RTC module	Microchip MCP7940N	Real-time Clock/Calendar (RTCC): Hours, minutes, Second, Day of week, Day, Month, Year	It is the RTC responsible for the timestamp of each of the monitoring stored in local memory.
		Oscillator for 32.768 kHz Crystals: Optimized for 6–9 pF crystal	
		On-Chip Digital Trimming/Calibration: ±1 PPM resolution ±129 PPM range	
Wireless Module	HoperF Electronics RF Module RFM69HWC	+13 dBm Power Output Capability	It is the radio used for wireless communication, with a range of +200 m in a straight line while utilizing low power consumption.
		High Sensitivity: down to −120 dBm at 1.2 kbps	
		High Selectivity: 16-tap FIR Channel Filter	
		Programmable Pout: −18 to +13 dBm in 1 dB steps	
		FSK Bit rates up to 300 kb/s	
		FSK, GFSK, MSK, GMSK and OOK modulations	
		115 dB + Dynamic Range RSSI	
		Packet engine with CRC-16, AES-128, 66-byte FIFO	

**Table 2:** Characteristics comprising the body of a node master.

Component	Model	Feature	Functionality
Raspberry Pi	Raspberry Pi model 3 B, Raspberry Pi Foundation, UK	Quad Core 1.2 GHz Broadcom BCM2837 64 bit CPU. RAM: 1 GB LPDDR2. GPIO: 40 pins. USB Ports: 4 × USB 2.0. Micro SD port for loading the operating system and storing data. Networking: 10/100 Ethernet, Wi-Fi, Bluetooth.	It serves as the central processing unit for data collection and processing.
GPS module	u-blox Neo 6M	Horizontal Position Accuracy: ±2.5 m Communication Protocol: NMEA, UBX Binary, RTCM Navigation Sensitivity: −161 dBm. Operating Voltage. Provides PPS signal.	It is the GPS receiver that provides the PPS signal used for synchronizing the monitoring system.
RTC module	Microchip MCP7940N	Real-time Clock/Calendar (RTCC): Hours, minutes, Second, Day of week, Day, Month, Year	The RTC is responsible for the timestamp of each of the monitoring stored data in local memory.
		Oscillator for 32.768 kHz Crystals: Optimized for 6–9 pF crystal	
		On-Chip Digital Trimming/Calibration: ±1 PPM resolution ±129 PPM range	
Wireless Module	HoperF Electronics RF Module RFM69HWC	+13 dBm Power Output Capability	The radio used for wireless communication, with a range of +200 m in a straight line while utilizing low power consumption.
		High Sensitivity: down to −120 dBm at 1.2 kbps	
		High Selectivity: 16-tap FIR Channel Filter	
		Programmable Pout: −18 to +13 dBm in 1 dB steps	
		FSK Bit rates up to 300 kb/s	
		FSK, GFSK, MSK, GMSK and OOK modulations	
		115 dB + Dynamic Range RSSI	
		Packet engine with CRC-16, AES-128, 66-byte FIFO	
		+13 dBm Power Output Capability	

**Table 3:** Statistical calculations of the fundamental frequencies of the I-beam with the impact on A.

Method	Mean	SD	CV
Sensor 1 RTC+PPS	15.707 Hz	0.53	3.372%
Sensor 2 RTC+PPS	15.591 Hz	0.473	3.034%
Sensor 1 Unsync	9.397 Hz	3.138	33.394%
Sensor 2 Unsync	15.108 Hz	0.255	1.686%

**Table 4:** Statistical calculations of the fundamental frequencies of the I-beam with the impact on B.

Method	Mean	SD	CV
Sensor 1 RTC+PPS	15.42 Hz	0.656	4.251%
Sensor 2 RTC+PPS	15.445 Hz	0.523	3.387%
Sensor 1 Unsync	15.488 Hz	0.261	1.684%
Sensor 2 Unsync	16.178 Hz	2.281	14.097%

## Data Availability

Data are contained within the article.
